# Clinical Significance of Measuring Global Hydroxymethylation of White Blood Cell DNA in Prostate Cancer: Comparison to PSA in a Pilot Exploratory Study

**DOI:** 10.3390/ijms18112465

**Published:** 2017-11-20

**Authors:** Alin Grelus, Dragos V. Nica, Imola Miklos, Valerica Belengeanu, Ioan Ioiart, Cristina Popescu

**Affiliations:** 1 Institute of Life Sciences, “Vasile Goldis” Western University of Arad, Str. Liviu Rebreanu 86, 310045 Arad, Romania; scmuro@gmail.com (A.G.); nicadragos@gmail.com (D.V.N.); miklosimola@gmail.com (I.M.); belvtim@yahoo.com (V.B.); ioiartioan@gmail.com (I.I.); 2 Arad County Emergency Clinical Hospital, Str. Andreny Karoly nr. 2–4, 310037 Arad, Romania; 3 Faculty of Pharmacy, “Vasile Goldis” Western University of Arad, Str. Liviu Rebreanu 86, 310045 Arad, Romania

**Keywords:** 5-hydroxymethylcytosine, prostate cancer, epigenetic biomarkers, white blood cells, prostate-specific antigen

## Abstract

This is the first study investigating the clinical relevance of 5-hydroxymethylcytosine (5hmC) in genomic DNA from white blood cells (WBC) in the context of prostate cancer (PCa) and other prostate pathologies. Using an enzyme-linked immunosorbent assay, we identified significantly different distributions of patients with low and elevated 5hmC content in WBC DNA across controls and patients with prostate cancer (PCa), atypical small acinar proliferation (ASAP), and benign prostatic hyperplasia (BPH). The measured values were within the normal range for most PCa patients, while the latter category was predominant for ASAP. We observed a wider heterogeneity in 5hmC content in all of the prostate pathologies analyzed when compared to the healthy age-matched controls. When compared to blood levels of prostate-specific antigen (PSA), this 5hmC-based biomarker had a lower performance in PCa detection than the use of a PSA cut-off of 2.5 nanograms per milliliter (ng/mL). Above this threshold, however, it delineated almost three quarters of PCa patients from controls and patients with other prostate pathologies. Overall, genome-wide 5hmC content of WBC DNA appears to be applicable for detecting non-cancerous prostate diseases, rather than PCa. Our results also suggest a potential clinical usefulness of complementing PSA as a PCa marker by the addition of a set of hydroxymethylation markers in the blood, but further studies are necessary to confirm these findings.

## 1. Introduction

The incidence of prostate diseases is expected to substantially increase during the next decades as the world’s ageing population continues to grow. Prostate cancer (PCa) and benign prostatic hyperplasia (BPH) are two high-profile prostate conditions that are encountered in men over 50 years of age [1]. It is estimated that the lifetime risk of developing PCa and BPH is one out of seven men and more than one out of two men, respectively [2,3]. To date, the prostate-specific antigen (PSA) blood test is the most commonly used test to help detect PCa, premalignant conditions, such as atypical small acinar proliferation (ASAP), and other prostate abnormalities. Convincing evidence supports that about two-thirds of PSA results are false-positives for PCa [4,5,6], which may lead to overdiagnosis, overtreatment, and adverse effects that the treatment may cause [7].

In this context, much emphasis has been put on developing and validating novel molecular biomarkers for PCa detection and prognosis. Increasing evidence for the role of epigenetic mechanisms in prostate development and disease processes has fostered research addressing the clinical utility of DNA methylation, histone modifications, and microRNAs as biomarkers for PCa and other prostate pathologies [8]. Methylation of the cytosine 5′ carbon (5-methylcytosine, 5mC) in CpG dinucleotides is a dynamic process, which is critical for transcriptional gene silencing and the repression of transposable elements [9]. Another important, yet poorly understood form of DNA methylation—5-hydroxymethylcytosine (5hmC)—is central to active DNA demethylation via the ten eleven translocation (TET) enzymes, and appears to be a hallmark that is associated with active gene transcription [10]. Most of the research to date investigating the applicability of these epigenetic marks as PCa biomarkers have focused on gene-specific DNA methylation in prostate tissue samples [11] and bodily fluids, including whole blood, plasma, serum, urine, and semen [12]. The few clinical investigations that examined global DNA methylation/hydroxymethylation in relation to PCa have used tumoral genomic DNA or circulating cell-free DNA as a source of DNA [13,14,15,16,17], with the exception of a very recent study using the DNA from white blood cells (WBC, syn. leukocytes) for this purpose [18]. This source of DNA provides investigators with a rich source of quality DNA, which is readily obtainable via non-invasive methods and is easy handleable for laboratory processing and clinical use [19]. Despite these advantages, no studies have examined the 5hmC content of WBC DNA in connection with PCa or any other prostate pathology type.

Here, we measured 5hmC by enzyme-linked immunosorbent assay (ELISA) in DNA from peripheral blood samples of individuals with BPH, ASAP, and PCa and in age-matched controls. Blood PSA was used as a reference biomarker to evaluate the performance of this epigenetic mark for discriminating PCa from normal prostate and other prostate conditions. In accordance with the recommendation of the National Comprehensive Cancer Network (NCCN), we used a PSA cut-off level of 2.5 nanograms per milliliter (ng/mL) [20]. In this pilot prospective study, we have also explored whether the use of 5hmC content of WBC DNA in conjunction with serum PSA could be used in the detection of PCa.

## 2. Results

**Figure 1:**
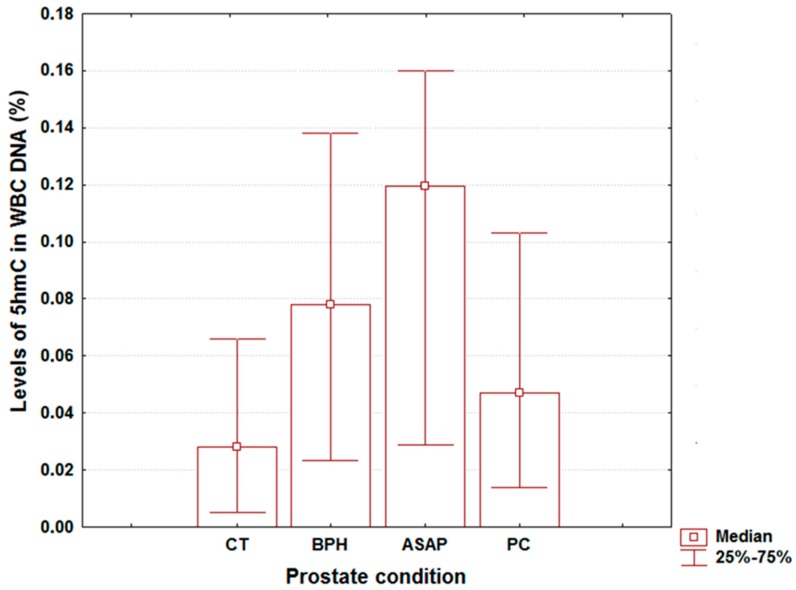
Measured values for 5hmC levels in white blood cells (WBC) DNA from patients with different prostate pathologies. Data are shown as medians with lower and upper quartiles (in paranthesis). CT, controls; BPH, benign prostatic hyperplasia; ASAP, atypical small acinar proliferation; PCa, prostate cancer.

The overall median values (with lower quartiles and upper quartiles) for age, global DNA hydroxymethylation, and PSA were 70 years (64, 75), 5.43 ng/mL (0.94, 13.00), and 0.061% (0.018, 0.124), respectively. Table 1 shows the medians for the first two variables across different prostate pathologies, whereas Figure 1 depicts the corresponding values for total 5hmC content in WBC DNA. The age data were similar among different groups (Kruskall-Wallis test, *p* = 0.161), attesting to the homogeneity of groups in terms of patient age.

The median values of the blood PSA levels tended to increase with the severity of prostate pathology (Table 1), with the PCa patients displaying the widest interquartile range (IQR). The measured values for 5hmC levels of WBC DNA in the PCa patients laid close to that observed in controls (Figure 1). This is in contrast with BPH and ASAP, in which case the medians were much higher when compared to healthy patients (Figure 1). Interestingly, the global WBC DNA hydroxymethylation revealed for all of the prostate conditions analyzed here a wider IQR than in the controls (Figure 1).

The distribution of DNA hydroxymethylation classes and PSA classes, as well as the association between these variables among patients with different prostate pathologies are presented in Table 2. We found significant differences in the distribution of patients with low and elevated 5hmC levels among the prostate pathologies investigated (chi2 test, *p* = 0.043). About two-thirds of PCa cases showed low DNA hydroxymethylation levels, whereas for ASAP, the patients with elevated 5hmC levels in WBC DNA were predominant (Table 2). Statistical analysis also revealed significant differences in the distribution of PSA classes across different prostate conditions (chi2 test, *p* < 0.001). Most healthy subjects and over half of the BPH patients had low blood PSA levels (Table 2). The ASAP patients displayed by far the highest prevalence of intermediate PSA levels (Table 2). In contrast, the highest percentage of patients with an increased blood PSA was observed for PCa while no such cases were detected in the control group (Table 2). Correlational analysis revealed no significant association between blood PSA levels and 5hmC content in genomic WBC DNA, irrespective of prostate pathology analyzed (Table 2).

Data for distribution of patients among different DNA hydroxymetylation classes and PSA classes are shown in the two middle columns as percentages with absolute values (in paranthesis). Associations corresponding to each prostate pathology were analyzed for total 5hmC content in WBC DNA, and serum PSA levels are presented in the right column as the values of the Spearman’s correlation coefficients with *p* values (in paranthesis). Marked values (*) denote significant association between these variables (* - *p* < 0.05). CT, controls; BPH, benign prostatic hyperplasia; ASAP, atypical small acinar proliferation; PCa, prostate cancer.

The performance of a PSA cut-off of 2.5 ng/mLin the detection of PCa was 96.30% in terms of sensitivity and 61.18% in terms of specificity. Stratification of patients based on the upper quartileof 5hmC level in controls allowed for a moderate sensitivity (66.67%) and low specificity (54.24%) in the diagnosis of this cancer type. However, when removing the patients with low blood PSA, the sensitivity remained similar (65.38%), whereas the specificity increased well above that observed when assessing the performance of PSA solely as a biomarker for the detection of PCa (69.28%).

## 3. Discussion

Here, we examine, for the first time, the clinical relevance of 5hmC levels in WBC DNA in the context of disturbed prostatic function, including PCa. This expands our knowledge on the connection between cancer and genomic (hydroxy)methylation of peripheral blood DNA, which until now was limited to 5mC data related to bladder, breast, colon, head, neck, stomach, and prostate cancer [18,21,22,23,24,25,26,27,28], and 5hmC data related to lung cancer and different types of blood cancer [29,30]. By using an ELISA assay, we were not able to determine 5hmC content at individual *CpG* sites. However, using our genome-wide hydroxymethylationmeasurements—describing the sum of all the cytosine hydroxymethylation events in WBC DNAs—we found significant differences in the distribution of patients with low and elevated 5hmC content among different prostatic pathologies. The amount of 5hmC in controls was consistent with that determined with an avidin-biotin ELISA system in similarly-aged healthy subjects [30]. The overall range of 5hmC levels in peripheral blood DNA measured in this study was comparable to those reported in two previous works when using the same ELISA-based approach for the quantitation of global leukocyte DNA hydroxymethylation, that is 0.02–0.25% of the total cytosine in the DNA [31,32].

Clinical investigations provide support for a complex interplay among inflammation, immune response, and prostate carcinogenesis [33,34,35]. Changes in the density of different subtypes of WBC are documented to occur in patients with disturbed prostatic function [33,34]. Moreover, genomic levels of 5mC can differ depending on WBC subtype [36]. Such findings render possible changes in genomic (hydroxy)methylation levels of peripheral blood DNA in response to distant prostate tissue alterations. However, the values measured here by ELISA were within the normal range for most PCa subjects. This is in contrast with the promising results that were obtained for lung cancer, in which case the presence of metastatic tumors was associated with a significant loss of 5hmC at CpGs in WBC DNA [30]. These data suggest that the usefulness of genomic hydroxymethylation of peripheral blood DNA for cancer detection may be related to the type of cancer and do not favour the use of this epigenetic mark as a single biomarker for PCa diagnostic.

Our results also provide scientists with the first data on global WBC DNA (hydroxy)methylation in BPH and ASAP. For both of these prostate pathologies, genomic 5hmC content in leukocyte DNA tended to be elevated when compared to controls and the PCa cases. The stage of cellular differentiation is directly associated with genome-wide DNA hydroxymethylation levels [37,38], and hence one possible explanation for such differences is that a new clone of circulating lymphocytes emerges in response to inflammation associated with BPH and ASAP [39] and modifies total 5hmC content of peripheral blood DNA. Because 5mC serve as the substrate for TET-mediated conversion to 5hmC, an alternative explanation is that these changes could simply reflect correlated changes in 5mC levels. Indeed, the only investigation linking WBC DNA to PCa has identified a reduced risk of PCa within five years of blood draw in patients with higher methylation of all CPGs of peripheral blood DNA [18].

We note here that the PCa patients displayed a broader range of 5hmC levels than controls. This increased heterogeneity of genomic hydroxymethylation of WBC DNA, which also occurred in BPH and ASAP, may reflect the altered expression of genes that are involved in the cellular polarity pathway (e.g., *BRSK2*, *STK11*, *FBF1*) or in regulating TETs expression (e.g., *TET1*, *TET2*), as has been already described in prostate tumors [16,40]. Overall, the clinical relevance of 5hmC content in WBC DNA as a single biomarker in the context of prostate appears to be related to the health status of this gland. That is, the high variability of 5hmC levels from peripheral blood DNA could indicate disturbed prostatic function, with elevated DNA hydroxymethylation being associated with an increased likelihood of patients having benign and potentially premalignant prostate pathologies.

Unlike our findings, genome-wide DNA hydroxymethylation in prostate tissues appears to be a sensitive biomarker for PCa detection. Application of dot-blot and immunohistochemistry (IHC) methodologies revealed marked changes in tumoral tissue samples when compared to normal prostate, especially a substantive loss of 5hmC in PCa [15,16,37,41,42,43]. A similar trend was reported for global DNA methylation in prostate tissues, as recently reviewed [44], but accumulating evidence suggest that total 5hmC content does not necessarily correlate with global 5mC content in normal human tissues [37,38], as well as in tumor tissue samples [45]. The magnitude of these changes is in contrast with the relatively small differences in median 5hmc levels of WBC DNA that are observed in the present study between PCa and controls. These discrepancies are highly unlikely to originate only from methodological issues [46,47] and inter-tissue differences in total 5hmC content [38,48]; rather, these differences may reflect the fact that the applicability of global DNA hydroxymethylation for PCa diagnosis is tissue-specific.

The lack of correlation between 5hmC levels in WBC DNA and blood PSA, indicates that, as expected, these two variables are independent biomarkers, which may reflect different molecular aspects underlying disturbed prostatic function. The overall trend towards the increasing PSA value with the severity of prostate pathology attests here to the clinical value of blood PSA for PCa screening and for delineating between different prostate pathologies [49,50]. The use of a PSA threshold of 2.5 ng/mL for men over 60 years had a high sensitivity, but a moderate specificity in the detection of PCa, which is in line with previous works investigating the performance of PSA for PCa diagnosis [51,52,53]. In contrast, WBC genome-wide DNA hydroxymethylation had a lower performance in the PCa screening.

However, when the patients with low levels of serum PSA were removed from the cohort, the specificity of 5hmC content in WBC DNA as a PCa biomarker improved notably and allowed us to discriminate this type of cancer from controls and other prostate conditions in almost three quarters of patients, while its sensitivity remained moderate. This finding deserves further consideration regarding the clinical usefulnes of complementing PSA as a marker of PCa presence by the addition of a set of hydroxymethylation markers in serum and/or stratifying patients with PCa based on total 5hmC content of peripheral blood DNA. Additional studies are necessary to shed light on the potential applicability of 5hmC-based blood biomarkers in the detection and prognosis of PCa. For example, locus-specific 5hmC content at the level of gene promoter regions and specific intronic and intergenic regions, such as *BARD1* signaling and steroid hormone receptor signaling [15], may be more specific and sensitive in the detection of PCa than genome-wide DNA hydroxymethylation. It will also be of interest to couple 5hmC-based blood biomarkers with highly specific and sensitive 5mC-based blood biomarkers for PCa, like the hypermethylation of glutathione-S-transferase (*GSTP1*) promoter [54], and subdivide PCa cases into distinct epigenetic classes that are based on these biomarkers. Since 5hmC is higher in neuronal cells [38], it will also be intriguing to determine whether this epigenetic mark occurs at higher amounts in neuroendocrine PCa. Furthermore, it would be important to understand the potential role for inflammation-related epigenetic changes in prostatic carcinogenesis and tumour progression [39]. Parallel measurements of levels of inflammatory biomarkers (e.g., C-reactive protein, interleukins) and DNA (hydroxy)methylation in the blood and prostate tissues in patients with different prostate pathologies should provide a strong basis for understanding such interactions.

We recognize that our pilot study has several potential limitations. First, it is possible that there might be chance findings or false positive findings given the moderate and unequal sample sizes. Yet, such experimental designs are commonly used in clinical pilot exploratory investigations [55,56] due to various reasons, such as cost, ethical, and patient availability [57]. In this case, the use of categorical arrays should provide scientists with a reliable statistical approach for continuous variables with skewed distribution and broad heterogeneity, such as the 5mC/5hmC data sets [39], because it simplifies statistical analysis and leads to an easy interpretation of results that are obtained [58]. Second, as already described for 5mC [42,59], DNA hydroxymenthylation may differ depending on WBC subtype and the distribution of WBC subtype may vary among individuals, which could possibly interfere with the results obtained. Third, we have hot examined the expression levels of key enzymes for 5hmC regulation, such as TETs or isocitrate dehydrogenases (IDHs) [37], which should be investigated in future studies. Finally, we cannot totally exclude actual epigenetic variation resulting from different levels of exposure to environmental factors (e.g., arsenic, lead, persistent organic pollutants, air pollution) or behavioural factors (e.g., smoking, alcohol consumption, physical activity, dietary folate). These factors may interfere with DNA (hydroxy)methylation in WBC [31,32,36], but they were not considered in this study.

## 4. Materials and Methods

### 4.1. Study Population

The present study is an exploratory pilot study that aimed to investigate the potential implications of genome-wide hydroxymethylation of peripheral blood DNA for clinical practice in patients with different prostate pathologies. To our knowledge, there are currently no published studies investigating the relevance of this blood-based marker for detecting prostate dysfunction in any (pre)clinical setting. Hence, we have chosen a heterogenous group of patients in order to gain information relevant of the potential scientific and clinical implications of this epigenetic mark in the context of prostate. This case-control study was performed after obtaining informed written consent from all of the participants and approval from the Human Research and Ethics Committee of the “Vasile Goldis” Western University of Arad (RUTE 2854 01.10.2015–01.10.2017, Ethic Approval no. 49/11 December 2015). We identified 77 patients with BPH, ASAP, and PCa, who attended a urology consult at the Urology Clinic of the Arad County Emergency Hospital between 15 June 2016 and 15 October 2016. Additionally, nine healthy age-matched patients were included in this study as controls. All of the patients were diagnosed after performing the PSA test in conjunction with digital rectal examination (DRE) and prostate biopsy. Samples of peripheral blood (3 mL) were collected prior to diagnosis and PSA levels in the blood were determined within 24 h after the visit. For all of the groups, the patients’ age was compiled from the hospital’s medical case-history record.

### 4.2. Genomic DNA Extraction

Human genomic DNA was extracted from fresh peripheral WBC using the QIAamp DNA Blood Maxi Kit (Qiagen, Hilden, Germany; Cat No. 51192) according to the manufacturer’sinstructions. Once extracted, the DNA was checked for quality (260/280 nm, NanoDrop-2000, Thermo Fisher Scientific Inc., Waltham, MA, USA) and stored at −80 °C for further analysis.

### 4.3. Quantification of Global DNA Methylation in WBC

The 5hmC content of genomic WBC DNA in patients with different prostate pathologies and control samples was determined by colorimetric ELISA with the Quest 5-hmC^TM^ DNA ELISA Kit (Zymo Research, Irvine, CA, USA; Cat No. D5426). This is the most cited ELISA-based global 5hmC quantification kit in the literature (over 60 citations according to Google Scholar), and provides scientists with a quick, cost-effective, and reliable alternative to chromatographic methods (e.g., LC-MS/MS, HPLC) that are used for this purpose, particularly for serial measurements, which was the case of our study. Assays were conducted in duplicate per each sample, according to the manufacturer’s instructions by loading 100 ng of DNA per well. To limit variation in measurements related to potential batch or procedural discrepancies, we have randomly rerun ELISA analysis for about a quarter of DNA samples. Optical density (OD) was red at 450 nanometers (nm) using a microplate Reader Stat Fax 4200 (Awareness Technology, Palm City, FL, USA). For absolute 5hmC quantification, a standard curve was obtained by plotting the various concentrations of the positive controls against the corresponding ODs.

### 4.4. Statistical Analysis

All statistical analyses were conducted using Statistica version 7 (StatSoft Inc., Tulsa, OK, USA). In all of the cases, a *p* value < 0.05 was considered significant. The inter-group homogeneity of age among different groups was checked using a Kruskall-Wallis test with tied ranks. To split the patients into low and elevated 5hmC groups, we defined the upper (75th) percentile of 5hmC quantities from controls as the cut-off point. Based on the absolute value of serum PSA, the men were stratified as having low PSA levels (up to 2.5 ng/mL), intermediate PSA levels (above 2.5 to 10 ng/mL), and high PSA levels (above 10 ng/mL), respectively. Chi2 tests were next applied on the distribution of PSA classes and 5hmC classes, respectively, across the prostate pathologies investigated, with the frequency observed in controls being used as a benchmark. Next, we examined the relationships between the blood PSA level and the total 5hmC content in peripheral blood DNA for each prostate pathology using Spearman’s correlations. Finally, we compared the performance of PSA and WBC genomic DNA hydroxymethylation as biomarkers for PCa detection.

## 5. Conclusions

Here, we investigated for the first time the potential clinical significance of 5-hydroxymethylcytosine in genomic DNA from white blood cells in the context of disturbed prostatic function. Genome-wide 5hmC levels were determined by enzyme-linked immunosorbent assay in patients with prostate cancer, atypical small acinar proliferation, benign prostatic hyperplasia, and age-matched controls. The measured values showed a wider heterogeneity in patients with prostate pathologies than in controls. Patients with low and elevated 5hmC content showed significantly different distributions across different prostate conditions. The distribution of these patients in prostate cancer was similar to that observed in healthy subjects, while for atypical small acinar proliferation, most patients had increased 5hmC levels. This 5hmC-based biomarker had a lower performance in the detection of prostate cancer in comparison with blood levels of prostate-specific antigen when using a cut-off point of 2.5 nanograms per milliliter. Above this threshold value, however, this biomarker allowed us to distinguish almost three-quarters of patients with prostate cancer from controls and those having other prostate pathologies. Our results do not favor the use of global hydroxymethylation of peripheral blood DNA for the detection of prostate cancer, but rather for indicating the presence of non-cancerous prostate diseases. The present findings also deserve to be expanded with regard to the potential clinical usefulnes of complementing PSA as a marker of PCa presence by the addition of a set of hydroxymethylation markers in the blood.

## Figures and Tables

**Table 1:** Measured values for age and prostate-specific antigen (PSA) levels in patients with different prostate pathologies.

Prostate Pathology	Age (Years)	PSA (ng/mL)
CT (control, *n* = 9)	63 (61, 68)	0.89 (0.29, 1.53)
BPH (*n* = 40)	70 (60, 75)	2.05 (0.52, 5.33)
ASAP (*n* = 10)	68 (62, 79)	7.61 (6.44, 9.39)
PCa (*n* = 30)	72 (67, 76)	34.01 (12.81, 116.02)

Data are shown as medians with lower and upper quartiles (in paranthesis). CT, controls; BPH, benign prostatic hyperplasia; ASAP, atypical small acinar proliferation; PCa, prostate cancer.

**Table 2:** Distribution of DNA hydroxymethylation and PSA classes and association between these variables among patients with different prostate pathologies.

Prostate Pathology	DNA Hydroxymethylation Class		PSA Class			5hmc-PSA Correlation
	Low	High	Low	Intermediate	High	
CT (*n* = 9)	77.78% (7)	22.22% (2)	88.89% (8)	11.11% (1)	0.00% (0)	0.18 (0.62)
BPH (*n* = 40)	42.50% (17)	57.50% (23)	60.00% (24)	35.00% (14)	5.00% (2)	0.22 (0.15)
ASAP (*n* = 10)	30.00% (3)	70.00% (7)	10.00% (1)	70.00% (7)	20.00% (2)	0.12 (0.73)
PCa (*n* = 30)	66.67% (18)	33.33% (9)	3.70% (1)	7.41% (2)	88.89% (4)	0.23 (0.24)
